# To treat or not to treat: comparison of different criteria used to determine whether weight loss is to be recommended

**DOI:** 10.1186/1475-2891-7-5

**Published:** 2008-01-29

**Authors:** Ottavia Colombo, Simona Villani, Giovanna Pinelli, Claudia Trentani, Maurizia Baldi, Orazio Tomarchio, Anna Tagliabue

**Affiliations:** 1 Department of Applied Health Sciences – University of Pavia, via A. Bassi 21, I-27100 Pavia, Italy; 2 IRCCS Salvatore Maugeri Foundation, via Maugeri, I-27100 Pavia, Italy

## Abstract

**Background:**

Excess body fat is a major risk factor for disease primarily due to its endocrine activity. In recent years several criteria have been introduced to evaluate this factor. Nevertheless, treatment need is currently assessed only on the basis of an individual's Body Mass Index (BMI), calculated as body weight (in kg) divided by height in m^2^. The aim of our study was to determine whether application of the BMI, compared to adiposity-based criteria, results in underestimation of the number of subjects needing lifestyle intervention.

**Methods:**

We compared treatment need based on BMI classification with four adiposity-based criteria: percentage body fat (%BF), considered both alone and in relation to metabolic syndrome risk (MS), waist circumference (WC), as an index of abdominal fat, and Body Fat Mass Index (BFMI, calculated as fat mass in kg divided by height in m^2^) in 63 volunteers (23 men and 40 women, aged 20 – 65 years).

**Results:**

According to the classification based on BMI, 6.3% of subjects were underweight, 52.4% were normal weight, 30.2% were overweight, and 11.1% were obese. Agreement between the BMI categories and the other classification criteria categories varied; the most notable discrepancy emerged in the underweight and overweight categories. BMI compared to almost all of the other adiposity-based criteria, identified a lower percentage of subjects for whom treatment would be recommended. In particular, the proportion of subjects for whom clinicians would strongly recommend weight loss on the basis of their BMI (11.1%) was significantly lower than those identified according to WC (25.4%, p = 0.004), %BF (28.6%, p = 0.003), and MS (33.9%, p = 0.002).

**Conclusion:**

The use of the BMI alone, as opposed to an assessment based on body composition, to identify individuals needing lifestyle intervention may lead to unfortunate misclassifications. Population-specific data on the relationships between body composition, morbidity, and mortality are needed to improve the diagnosis and treatment of at-risk individuals.

## Background

It is generally accepted that several major diseases are related to overweight and obesity. These include metabolic syndrome and type 2 diabetes mellitus, cardiovascular diseases, some tumours, gallbladder diseases, non-alcoholic steatohepatitis, sleep apnoea and osteoarthritis [1-6]. The endocrine activity of adipose tissue is strongly implicated in most of these diseases [7,8]. Therefore, excess body fat rather than excess body weight is detrimental to health; hence, paradoxically, metabolically obese, normal weight individuals, not deemed obese on the basis of height and weight parameters, were found to be hyperinsulinaemic, insulin-resistant, predisposed to type 2 diabetes mellitus, hypertriglyceridaemic, or to have premature coronary heart disease; these individuals responded favourably to caloric restriction [9-11].

Health professionals should assess patients on the basis of their body composition rather than their body weight [12]. Thus, new clinical criteria have been introduced to evaluate body adiposity, such as percentage body fat (%BF), considered both alone [13] and in relation to metabolic syndrome risk [14], and waist circumference, as an index of abdominal fat [1,15]. Recently, the Body Fat Mass Index (BFMI, calculated as fat mass in kg divided by height in m^2^) was introduced, in nutritional assessment, as an additional element for assessing adiposity [16-18].

Nevertheless, treatment need is currently assessed on the basis of an individual's Body Mass Index (BMI), calculated as their body weight (in kg) divided by their height in m^2 ^[1].

The aim of our study was to determine whether application of the BMI, compared to adiposity-based criteria, results in underestimation of the number of subjects needing a lifestyle intervention.

## Methods

### Sample

A sample of 63 volunteers (all white, 23 men and 40 women, aged 20–65 years), was recruited. To be included in the study they had to have a good health status, a sedentary lifestyle, and could not be on a low-calorie diet or on drug therapy for acute or chronic illnesses. All the recruited subjects attended the Human Nutrition and Eating Disorders Research Centre, University of Pavia, between January 2004 and January 2005 to undergo a nutritional assessment and body composition analysis; the diagnostic radiology examinations were carried out at the IRCCS Salvatore Maugeri Foundation, Pavia.

### Study design

The subjects attended the dietology outpatient clinic early in the morning after an overnight fast. On arrival, they emptied their bladders. Anthropometric measurements were taken. Body composition was assessed by means of bioimpedance analysis (BIA) and then dual-energy X-ray absorptiometry (DXA). Later in the morning, when the examinations were complete, the subjects were allowed to eat.

### Anthropometric measurements

The subjects, wearing minimal clothing, were weighed to the nearest 0.1 kg using a balance beam scale equipped with a stadiometer. Their height, standing barefoot, was measured to the nearest 0.5 cm. Their BMI was calculated in the standard way: weight in kg divided by height in m^2^. The subjects were then classified into four groups according to the WHO BMI cut-offs [1] :

A) BMI: "underweight" : BMI < 18.5 kg/m^2^

"normal weight": BMI = 18.5 – 24.9 kg/m^2^

"overweight" : BMI = 25 – 29.9 kg/m^2^

"obese" : BMI ≥ 30 kg/m^2^

Waist circumference was measured to the nearest 0.1 cm with a measuring tape placed at the midpoint between the lower border of the ribs and the upper border of the pelvis. The subjects were then divided, according to gender-specific waist circumference values, into three categories denoting risk of metabolic complications [1] :

B) waist circumference as an index of abdominal fat:

"not increased" : < 80 cm females, < 94 cm males

"increased" : 80–87.9 cm females, 94–101.9 cm males

"substantially increased" : ≥ 88 cm females, ≥ 102 cm males

### Body composition assessment

To reduce methodological biases when classifying subjects according to adiposity-based criteria, we used the same methods reported by the appropriate reference studies: DXA to measure %BF (C) [13] ; and BIA to assess both metabolic syndrome risk (D) [14] and BFMI (E) [16].

DXA was performed using a Norland RX-26 scanner (Norland Corp., W, USA), which automatically gave a %BF reading. BIA was performed using a Human Im Scan device (Dietosystem, Milan, Italy); %BF was calculated using Deurenberg's formula [19]. The subjects were then classified according to different criteria:

C) %BF: the subjects were classified according to %BF cut-offs calculated by Gallagher et al. for the white subgroup of their study population [13] (Table 1).

**Table 1:** Percentage body fat cut-offs for white people, proposed by Gallagher et al. [13].

**BMI (kg/m^2^)**	**body fat (% weight)**	
	
	Men	Women
20–39 y		
18.5	8	21
25.0	21	33
30.0	26	39

40–59 y		
18.5	11	23
25.0	23	35
30.0	29	41

60–79 y		
18.5	13	25
25.0	25	38
30.0	31	43

D) Metabolic syndrome risk: the subjects were classified on the basis of %BF cut-offs calculated by Zhu in the white subgroup of the American population that took part in NHANES III [14] (Table 2).

**Table 2:** Percentage body fat thresholds related to metabolic syndrome risk proposed by Zhu et al. for white people [14].

**BMI (kg/m^2^)**	**body fat (% weight)**	
	
	Men	Women
18.5	11.0	22.5
25.0	21.2	30.8
30.0	29.1	37.2

E) Body Fat Mass Index: the subjects were classified according to the BFMI cut-offs calculated by Kyle on the basis of a very large sample of white men and women living in Switzerland [16] (Table 3).

**Table 3:** Body Fat Mass Index (BFMI) cut-offs for healthy white adults, proposed by Kyle et al. [16].

**BMI (kg/m^2^)**	**BFMI (kg/m^2^)**	
	
	Men	Women
18.5	1.8	3.9
25.0	5.2	8.2
30.0	8.3	11.8

To classify the subjects for whom weight loss would be recommended or strongly recommended (for each criterion), we applied the following cut-offs (also see Tables 1, 2, 3).

Weight loss recommended:

(A) BMI ≥ 25 kg/m^2^, in both women and men;

(B) waist circumference ≥ 80 cm in women and ≥ 94 cm in men;

(C) total body fat, expressed as percentage body fat: %BF ≥ 33% in women and ≥ 21% in men in the 20–39 years age group, %BF ≥ 35% in women and ≥ 23% in men aged 40–59 years, %BF ≥ 38% in women and ≥ 25% in men aged 60–79 years;

(D) metabolic syndrome risk: %BF ≥ 30.8% in women and ≥ 21.2% in men;

(E) BFMI ≥ 8.2 kg/m^2 ^in women and ≥ 5.2 kg/m^2 ^in men ("overfat").

Weight loss strongly recommended:

(A) BMI ≥ 30 kg/m^2 ^in women and men;

(B) waist circumference ≥ 88 cm in women and ≥ 102 cm in men;

(C) total body fat, expressed as percentage body fat: %BF ≥ 39% in women and ≥ 26% in men in the 20–39 years age group, %BF ≥ 41% in women and ≥ 29% in men aged 40–59 years, %BF ≥ 43% in women and ≥ 31% in men aged 60–79 years;

(D) metabolic syndrome risk: %BF ≥ 37.2% in women and ≥ 29.1% in men;

(E) BFMI ≥ 11.8 kg/m^2 ^in women and ≥ 8.3 kg/m^2 ^in men (severely "overfat").

### Statistical analysis

Sex-related differences in anthropometric measurements and body fat indices were tested using unpaired t-tests or χ^2^-tests. Statistical significance was defined as p < 0.05. Agreement between BMI and abdominal fat, %BF, metabolic syndrome risk, and BFMI was evaluated using Kendall's Tau-b test. McNemar's test was applied to evaluate whether the percentage of subjects in whom weight loss was deemed necessary on the basis of BMI was equal to the percentages identified on the basis of the other body composition assessments; similarly, the percentage of subjects whose BMI would prompt a strong recommendation to lose weight was compared to the percentages of subjects in whom application of each of the other adiposity classification criteria also revealed a definite need to lose weight. All analyses were carried out with the statistical software program SPSS, version 13.0.

### Statement of ethics

We certify that this research complied fully with all applicable institutional and governmental regulations concerning the ethical use of human volunteers and with the terms of the Helsinki Declaration. The University of Pavia ethics committee approved the study protocol, and all the recruited subjects gave their written informed consent to take part.

## Results

The women enrolled in the study were slightly younger than the men (38.5 ± 14 years vs 39.2 ± 13.3 years), but this difference was not statistically significant (*t *= 0.201, p = 0.841).

On average, the men recorded higher body weight, waist circumference and BFMI values than the women, while the women had a higher %BF and metabolic syndrome risk (Table 4). Body fat percentage measured by DXA was significantly linearly related to that measured by BIA (*r*_Pearson _= 0.827, p < 0.0001).

**Table 4:** Summary of statistics by sex (mean values and standard deviations in square brackets).

	**n**	**Weight (kg)**	**BMI (kg/m^2^)**	**%BF**	**MS**	**WC (cm)**	**BFMI (kg/m^2^)**
Men	23	75.3 [11.2]	24.7 [3.9]	21.5 [8.6]	24.2 [7.6]	89.5 [10.8]	6.2 [2.8]
Women	40	62.0 [11.0]	23.8 [4.2]	34.8 [8.4]	32.6 [9.1]	80.8 [12.2]	8.0 [3.3]
**Test and p-value**		t = 4.59 p < 0.0001	t = 0.78 p = 0.438	t = -6.00 p < 0.0001	t = -3.52 p < 0.001	t = 2.83 p = 0.006	t = -2.01 p = 0.049

*%BF *= total body fat, expressed as percentage body fat; *MS *= metabolic syndrome risk: %BF related to the risk of developing metabolic syndrome; *WC *= waist circumference, as an index of abdominal fat; *BFMI *= Body Fat Mass Index

According to the classification based on BMI, 6.3% of subjects were underweight, 52.4% were normal weight, 30.2% were overweight, and 11.1% were obese. Abdominal adiposity was not increased in 63.5% of subjects, increased in 11.1%, and substantially increased in 25.4%. Total body fat, considered alone, was low in 3.2% of subjects, normal in 49.2%, increased in 19.0%, and high in 28.6%. Metabolic syndrome risk related to %BF was low in 7.1% of subjects, normal in 37.5%, increased in 21.4%, and high in 33.9%. BFMI was low in 3.6% of subjects, normal in 42.9%, increased in 32.1%, and high in 21.4%.

Metabolic syndrome risk was the only criterion to show significant gender-related differences (χ^2 ^= 9.430; p = 0.024): greater percentages of the women compared to the men showed a high metabolic syndrome risk (37.1% vs 28.6%), and a low and normal metabolic syndrome risk (8.6% vs 4.8% and 45.7% vs 23.8%, respectively); conversely, a greater proportion of the men had an increased metabolic syndrome risk (42.9% vs 8.6%).

Agreement between the BMI categories and the other classification criteria categories varied (Table 5). The most notable discrepancy emerged in the underweight and overweight categories. Of the subjects classed as underweight on the basis of BMI, 75% had normal %BF and metabolic syndrome risk values, and none had an increased waist circumference. In the BMI normal weight subjects, increased abdominal fat was found in 9.1%, increased %BF in 15.2%, and increased metabolic syndrome risk in 20%; a high %BF was found in 6.1% of the normal weight subjects and a high metabolic syndrome risk in 16.7%. In the overweight subjects, a marked discrepancy emerged between the BMI and the adiposity indices: more than 50% of the subjects had a high %BF and metabolic syndrome risk and just under 50% high abdominal fat. In the subjects rated as obese on the basis of their BMI, there was good agreement between all the criteria.

**Table 5:** Agreement between the BMI categories and the other classification criteria categories expressed as percentage values. The values in bold represent the main diagonal of the agreement matrix.

**Other classification criteria categories**	**Body Mass Index**			
	
	underweight	normal weight	**overweight**	**obese**
low				
*%BF*	***25.0***	3.0	-	-
*MS*	***25.0***	10.0	-	-
*BFMI*	***25.0***	3.3	-	-
normal				
*%BF*	75.0	***75.8***	15.8	-
*MS*	75.0	***53.3***	6.7	14.3
*WC*	100.0	***90.9***	31.6	-
*BFMI*	75.0	***66.7***	6.7	-
**increased**				
*%BF*	-	15.2	***31.6***	14.3
*MS*	-	20.0	***40.0***	-
*WC*	-	9.1	***21.1***	-
*BFMI*	-	30.0	***53.3***	14.3
**high**				
*%BF*	-	6.1	52.6	***85.7***
*MS*	-	16.7	53.3	***85.7***
*WC*	-	-	47.4	***100.0***
*BFMI*	-	-	40.0	***85.7***

*%BF *= total body fat, expressed as percentage body fat; *MS *= metabolic syndrome risk: %BF related to the risk of developing metabolic syndrome; *WC *= waist circumference, as an index of abdominal fat; *BFMI *= Body Fat Mass Index

Comparing the BMI with the BFMI categories, 75% of the underweight subjects had a normal fat status. In the BMI normal weight category, 30% of the subjects were "overfat" according to the BFMI, while in the overweight category only 6.7% of subjects had a normal fat status and 40% had a severely "overfat" status. There was good agreement between BMI and BFMI in the obese subjects. Overall, the BMI showed good agreement with BFMI (tau-b Kendall = 0.722, p < 0.001) and waist circumference (abdominal fat) (tau-b Kendall = 0.704, p < 0.001). The level of agreement between the various BMI categories and the %BF and metabolic syndrome risk categories was moderate (tau-b Kendall = 0.672, p < 0.001 and tau-b Kendall = 0.563, p < 0.001 respectively).

**Figure 1:**
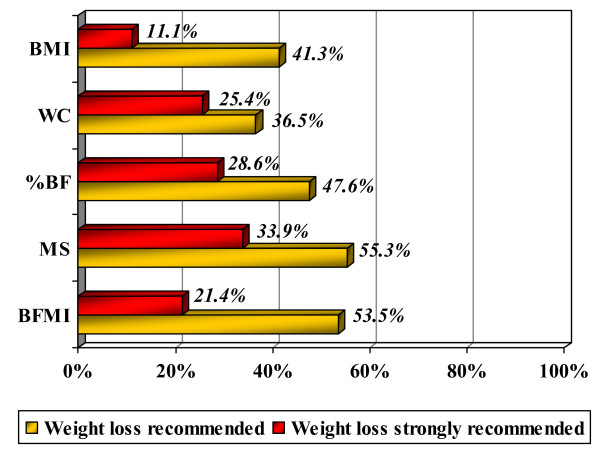
**Weight loss treatment recommendations for each criterion**. Percentage of subjects for whom weight loss would be recommended, including those for whom it would be strongly recommended, on the basis of the different criteria: BMI (Body Mass Index), WC (waist circumference, as an index of abdominal fat), %BF (total body fat, expressed as percentage body fat), MS (metabolic syndrome risk: %BF related to the risk of developing metabolic syndrome), BFMI (Body Fat Mass Index)

The percentages of subjects for whom weight loss treatment would be recommended and strongly recommended, on the basis of each criterion, are summarised in figure 1. The proportion of subjects for whom clinicians would recommend weight loss on the basis of their BMI (41.3%) was not significantly different from the proportions in whom it would be recommended on the basis of %BF (p = 0.344) and abdominal fat (p = 0.508). Conversely, a significant difference emerged in relation to the criteria metabolic syndrome risk (p = 0.022) and BFMI (p = 0.021). The picture changes when analysing the proportion of subjects for whom weight loss would be strongly recommended, with the proportion identified on the basis of BMI (11.1%) differing significantly from the proportion identified by abdominal fat (p = 0.004), %BF (p = 0.003), and metabolic syndrome risk (p = 0.002). Instead, no significant difference was found between the proportions of subjects whom clinicians, on the basis of BMI versus BFMI, would be strongly urged to lose weight (p = 0.125).

## Discussion

Obesity is a metabolic disorder characterised by excess body fat, which is an important risk factor for disease, not only because it is a volume-filling organ, but primarily due to the endocrine activity of adipose tissue [7,8,20].

Although the BMI is easily calculated and can be readily used in population studies, it does not discriminate between fat mass and fat-free mass, or reflect the fat mass distribution in the body [21-25]. In our study, the BMI compared to almost all of the other criteria, based on adiposity, identified a lower percentage of subjects for whom treatment would be recommended and strongly recommended. In particular, the difference was statistically significant when the BMI was compared to metabolic syndrome risk. These findings suggest that a certain proportion of subjects classified as normal weight on the basis of their BMI would not be recommended for treatment even though they harbour excess body fat which could have clinical and metabolic consequences. This is also true if we compare the BMI to the BFMI, the latter an index that denotes the amount of body fat in relation to stature. Indeed, the BFMI, which can be considered a qualitative evaluation of BMI, can result in a better clinical assessment of subjects. From a clinical point of view, basing an individual's treatment needs on their BMI can place at risk those subjects who, on the basis of their metabolic syndrome risk, %BF, and waist circumference, would be strongly advised to undergo weight-loss treatment.

As regards the underweight subjects, a large proportion of them had normal (not low) %BF, waist circumference, metabolic syndrome risk, and BFMI values. However, in these subjects, the misclassification does not have important clinical consequences, since treatment would not, on the basis of any of the criteria, be recommended for any of them.

Thus, our data confirm that indirect estimates of body composition (i.e. BMI) are useful for groups but unreliable in individuals [26]. Furthermore, our data also underline the importance of discriminating between lean mass and fat mass, and of relating these parameters to body height, in order to obtain a better nutritional assessment. In addition, the clinical consequences of an altered body composition should be taken into account. The use of the BMI alone to evaluate overweight and obese individuals leads to undesirable misclassifications. Of note, our study sample consisted of people with a sedentary lifestyle, which may predispose them to increased adiposity even before an increase in body weight becomes evident; this implies that normal weight subjects can be at risk of excessive adiposity.

Thus, there is a need to replace the BMI or to supplement it with other diagnostic criteria, in particular, ones that focus on body adiposity, considered both alone and with regard to its distribution. If confirmed by data from larger studies, our results highlight the need to investigate the clinical consequences of excess body fat in normal weight subjects. Several studies have focused on the relationship between the BMI and morbidity and mortality [2,3,27]. Unfortunately, to date, few studies have focused on the relationship between %BF and morbidity and mortality, and those that have been conducted were restricted to specific ethnic groups [14,16,28-31]. Thus, precise %BF cut-offs that can be used in clinical settings to evaluate an individual's health remain to be determined.

Since %BF has important clinical consequences, and treatment costs and drop-out rates among "overfat" subjects are high, there is clearly a pressing need for precise and unambiguous guidelines. In particular, %BF cut-offs should be defined that can be used in addition to the BMI and waist circumference values already published for the diagnosis and treatment of at-risk subjects [1].

## Conclusion

The use of the BMI alone, as opposed to an assessment based on body composition, to identify individuals needing lifestyle intervention may lead to unfortunate misclassifications. Population-specific data on the relationships between body composition, morbidity, and mortality are needed to improve the diagnosis and treatment of at-risk individuals.

## Competing interests

The author(s) declare that they have no competing interests.

## Authors' contributions

OC: conception and design of the study; generation, collection and assembly of data; interpretation of data; drafting of the manuscript. SV: analysis and interpretation of data. GP: conception and design of the study; generation, collection and assembly of data. CT: conception and design of the study. MT: collection, assembly and interpretation of data. OT: collection, assembly and interpretation of data. AT: conception and design of the study; generation, collection and assembly of data; interpretation of data; drafting of the manuscript. All the authors have read and approved the final manuscript.
